# Synthesis, characterization and irritant effects of nonivamide irritant riot control agent based on ionic liquids

**DOI:** 10.3389/fchem.2024.1508396

**Published:** 2024-11-25

**Authors:** Weiting Ma, Hongying Wang, Zhenxiong Wang, Dong Chen, Ling Yuan, Liang Qin, Zongshu Mei

**Affiliations:** State key Laboratory of NBC Protection for Civilian, Beijing, China

**Keywords:** nonivamide, riot control agent, ionic liquid, solubility, irritant effects

## Abstract

Active Pharmaceutical Ingredients-Ionic liquids (API-ILs) can increase drug solubility and bioavailability of solid drugs without changing the structure of drug molecules. In the present work, nonivamide (pelargonic acid vanillylamide, PAVA) was used as the active drug and choline (Ch) and citric acid (CA) were selected as the ions to prepare PAVA–based ionic liquid ([Ch][PAVA] and [PAVA]_3_ [CA]), respectively. The characterization and physical properties of [Ch][PAVA] and [PAVA]_3_ [CA], such as FT–IR spectra, 1H NMR spectra, thermal stability and hydrophilicity, were investigated. And the irritant effects of the PAVA–based ionic liquids were measured by the animal irritation experiment. [Ch][PAVA] has higher decomposition temperatures, better hydrophilicity and comparable irritant effects. The results show that the PAVA–based ILs synthesized in this study may potentially serve as a promising novel riot control agent.

## 1 Introduction

Nonivamide (PAVA; CAS registry number: 2444–46–4; Figure 1), known as synthetic capsaicin, is a capsaicinoid that exhibits various biological and pharmacological effects and has a wide range of applications in medicine (Constant et al., 1996; Rottke et al., 2014; Heck et al., 2017), health supplements (Rosa et al., 2009), pesticides (Kong et al., 2013), especially riot control agents (Reilly et al., 2001; Patocka and Kuca, 2011; Satpute et al., 2018; Mörén et al., 2024). PAVA exerts strong irritant effects on the eyes and upper respiratory tract. Upon contact with the human body, it immediately causes lacrimation, involuntary eye closure, coughing, sneezing, and chest pain, resulting in temporary incapacitation. Thus, the novel irritant has been widely used as an effective and safe incapacitating agent against violence by law enforcement agencies and police departments in many countries around the world (Reilly et al., 2001; Bettencourt da Silva et al., 2014; Wang et al., 2017; Antonio et al., 2019; Zhai and Cui, 2021).

**FIGURE 1:**
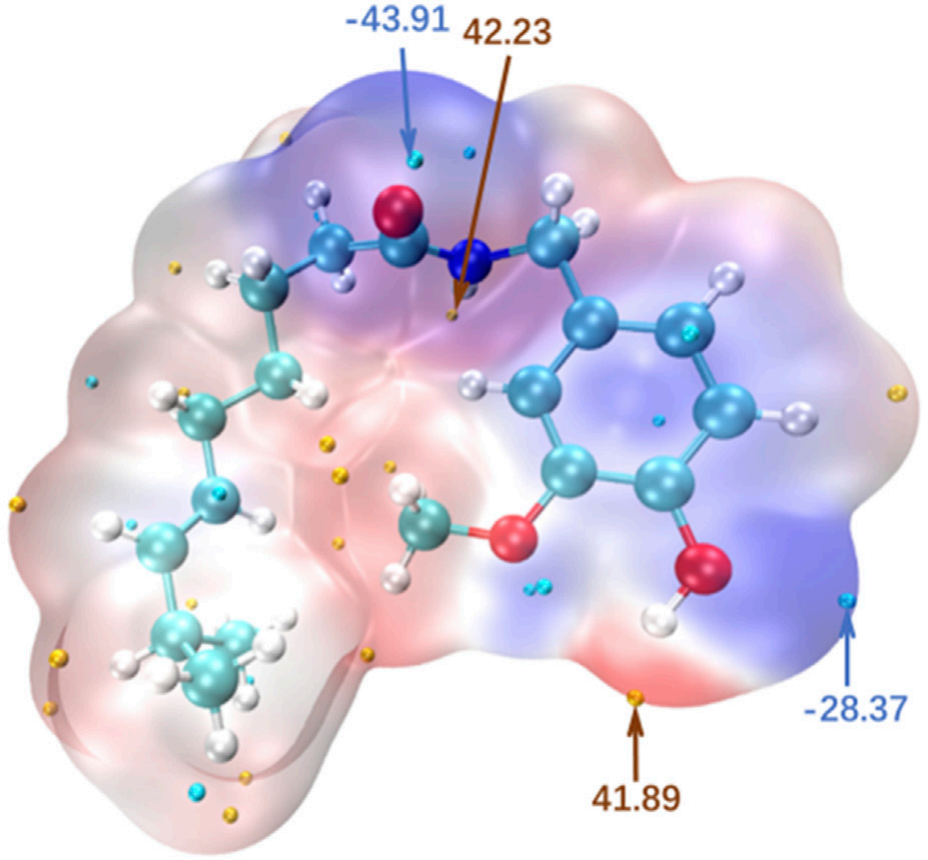
Molecular structure of PAVA.

Capsaicin–based incapacitating agents are typically deployed in two forms, namely liquid aerosol or ultrafine solid powder (Reilly et al., 2001; Luo and Wang, 2011; Bettencourt da Silva et al., 2014; Zhai and Cui, 2021). However, PAVA has extremely low hydrophilicity and can be considered practically insoluble in water (Lei et al., 2010; Yan et al., 2012; Cao et al., 2014), and the way for its application in tar gas liquid is adding considerable amounts of organic solubilizers and surfactants to boost the solubility of PAVA (Reilly et al., 2001; Bettencourt da Silva et al., 2014; Wang et al., 2017). The resultant has several drawbacks such as high production cost, low degree of environmental friendliness, and other environmental consequences that are difficult to address. Therefore, enhancing the hydrophilicity of capsaicinoids is a key technical hurdle that must be overcome for the preparation of environment–friendly, high–concentration, water–based formulations of PAVA.

Ionic liquids (ILs) are “designer liquids” composed entirely of organic cations and organic/inorganic anions (Zhang et al., 2010; Hu and Peng, 2014; Sosnowska et al., 2014; Mir et al., 2024). ILs are classified as green chemical solvents owing to characteristics such as flame retardancy, non–volatility, good chemical and thermal stability, and environmental friendliness (Sosnowska et al., 2014; Qu et al., 2022). In recent years, API-ILs have been regarded as powerful yet safe tools that can assist in overcoming the issues of low solubility and poor bioavailability in insoluble drugs (Ford et al., 2020; Lu et al., 2021; Gao and Shu, 2023; Shamshina and Rogers, 2023; Zhuo et al., 2024).

In this study, highly water–soluble choline cations ([Ch]^+^) were introduced into the IL molecules of PAVA to improve the hydrophilicity of PAVA via electrostatic and hydrogen bond interactions. Choline cations were selected as the ideal cations due to their superb hydrophilicity, permeability, and biocompatibility. Moreover, choline is generally recognized as safe (GRAS) by the United States Food and Drug Administration (USFDA (Sosnowska et al., 2014)). In addition, IL synthesis was also performed with citric acid (CA) and PAVA to compare the effects on the hydrophilicity of PAVA. Compared with PAVA, the solubility of [Ch][PAVA] was significantly increased. Furthermore, diluted solutions of the synthesized ILs exhibited irritant effects comparable to those of PAVA and capsaicin. Therefore, the PAVA–based ILs synthesized in this study may potentially serve as a promising novel irritant material.

## 2 Materials and methods

### 2.1 Reagents

The following reagents were used: PAVA, >98% purity (Shanghai yuanye Bio-Technology Co., Ltd., China); ethanol, analytical reagent (AR) grade (Beijing Chemical Works, China); choline hydroxide (50 wt% aqueous solution) and CA (Shanghai Macklin Biochemical Co., Ltd., China); distilled water (Hangzhou Wahaha Group Co., Ltd., China); Capsaicin, containing 60% capsaicin and 35% dihydrocapsaicin as active ingredients (Tianjin Heowns Biochemical Co., Ltd., China).

### 2.2 Animals

Dunkin Hartley guinea pigs (50%/50% male and female) weighing 200–300 g were purchased from Huafukang Beijing Bioscience Co., Ltd (Beijing, China). The guinea pigs were provided with an appropriate growth environment with adequate water and food. All animal experimentation was performed hewing to the Guidelines for the Care and Use of Experimental Animals, and was authorized by the State Key Laboratory of NBC Protection for Civilians under permission No. LAE-2023–03–002.

### 2.3 Synthesis of [Ch][PAVA] and [PAVA]_3_ [CA]

Synthesis of the [Ch][PAVA] and [PAVA]_3_ [CA] were performed as previously reported (Lu et al., 2021). PAVA powder (29.34 g, 0.1 mol) was dissolved in anhydrous ethanol (30 g) at about 30°C. Under conditions of vigorous stirring 1,000 rpm and 30°C, an equivalent number of moles of choline hydroxide (50 wt% aqueous solution) or an one-third number of moles of CA (20 wt% aqueous solution) was added to the PAVA–ethanol system and reacted under continuous stirring at room temperature for 24 h. Subsequently, ethanol and water were removed from the solution by rotary evaporation for 3 h at 65°C, and a brownish–yellow viscous IL ([Ch][PAVA] or [PAVA]_3_ [CA]) was obtained.

### 2.4 Characterization of prepared compounds

#### 2.4.1 Fourier transform infrared (FT–IR) spectra

Fourier transform infrared (FT–IR) spectra were recorded on a Mettler Toledo Nicolet 6700 FT–IR spectrometer with a wavelength range of 400–4,000 cm^−1^.

#### 2.4.2 ^1^H nuclear magnetic resonance (NMR) spectra

^1^H nuclear magnetic resonance (NMR) spectra were collected in Bruker 600 MHz NMR spectrometer, with DMSO–d_6_ and D_2_O separately used as solvents.

#### 2.4.3 Elemental analysis

Elemental analysis were measured using a elementar analyzer UNICUBE, with measurements performed using O_2_ atmosphere at 1,000°C for elements CHSN and He atmosphere for element O.

#### 2.4.4 Thermogravimetric analysis

Thermogravimetric analysis (TGA) spectra were measured using a Mettler Toledo thermogravimetric analyzer/differential scanning calorimeter (TGA/DSC) 2, with measurements performed using a heating rate of 10°C min^−1^ and in an air atmosphere.

#### 2.4.5 Contact angles measurement

Contact angles were measured using a Dataphysics OCA20 contact angle system. Each sample was first preheated in a 70°C water bath for the enhancement of fluidity, and the preheated sample was subsequently added dropwise onto a microscopic slide and subjected to contact angle measurement after cooling to room temperature.

#### 2.4.6 Irritation experiment

The degree of intensity of the irritant reactions of guinea pigs to different samples of identical concentration was measured to determine the strength of irritant effects. Experimental animals were placed in a viewable container. Identical volumes of each sample solution were separately sprayed onto the face of each animal, and the total number of face–scratching episodes over a fixed duration was recorded to observe the irritant responses of the animals (Szolcsányi and JancsóGábor, 1975). To compare the irritant effects of different irritant agents, 10% ethanol solutions of [Ch][PAVA], [PAVA]_3_ [CA], PAVA, and natural capsaicin (caps) were formulated for a comparative experiment performed on 16 guinea pigs (equal numbers of males and females) randomly divided into four groups.

## 3 Results and discussion

### 3.1 Characterization of the prepared compounds

**FIGURE 2:**
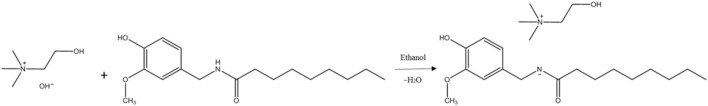
Pathway of [Ch][PAVA] synthesis.

In the present study, the [Ch][PAVA] IL was synthesized by simple mixing of equivalent numbers of moles of PAVA and choline hydroxide in ethanol solvent. Figure 2 shows the pathway of [Ch][PAVA] synthesis.

#### 3.1.1 FT–IR characterization of the prepared compounds

**FIGURE 3:**
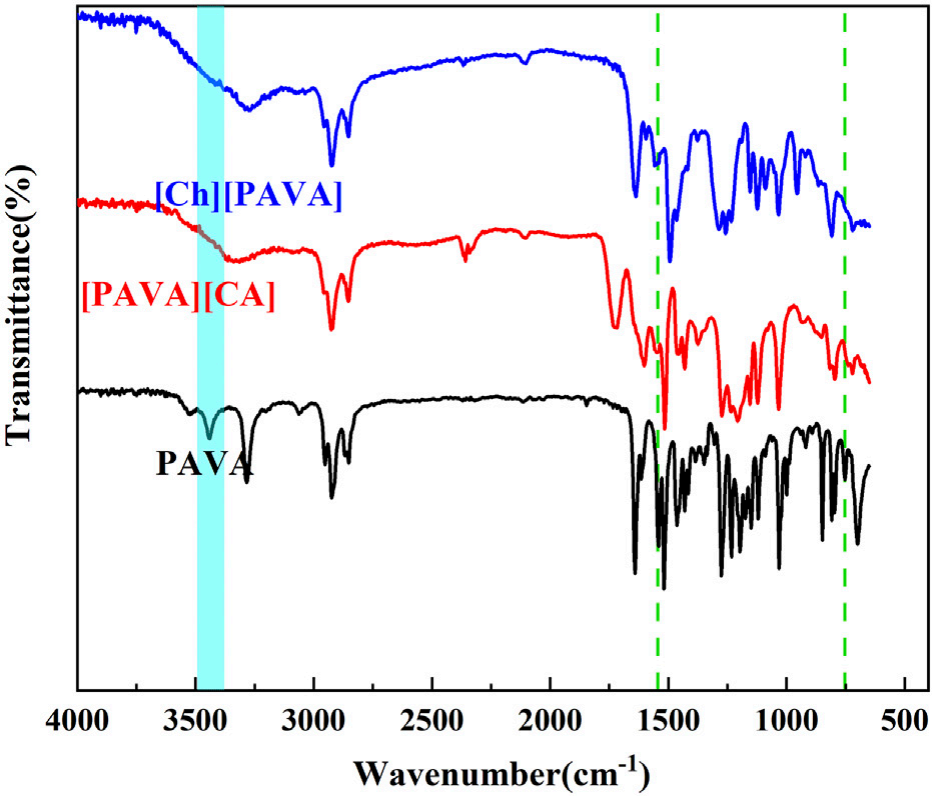
IR spectra of [Ch][PAVA], [PAVA]_3_ [CA] and PAVA.

The IR spectra (Figure 3) show the presence of the characteristic peak of the NH group of secondary amides in PAVA at 3,441 cm^−1^ and fingerprint peaks at 1,531 cm^−1^ and 752 cm^−1^. However, all three peaks were not observed in the IR spectrum of [Ch][PAVA] and [PAVA]_3_ [CA]. This is attributed to the fact that the NH group of secondary amides in PAVA is highly susceptible to replacement reactions and thus readily reacted during the IL synthesis process (see Figure 2). The sharp peak belonging to the phenolic hydroxyl group in PAVA at 3,283 cm^−1^ became wider in [Ch][PAVA] and [PAVA]_3_ [CA], possibly due to the formation of hydrogen bonds between phenolic hydroxyl groups and cations. Peak locations in the fingerprint region of [Ch][PAVA] were shifted compared with those of PAVA, which may be caused by the substantial influence of cation-anion interactions on functional groups.

#### 3.1.2 NMR characterization of the prepared compounds

**FIGURE 4:**
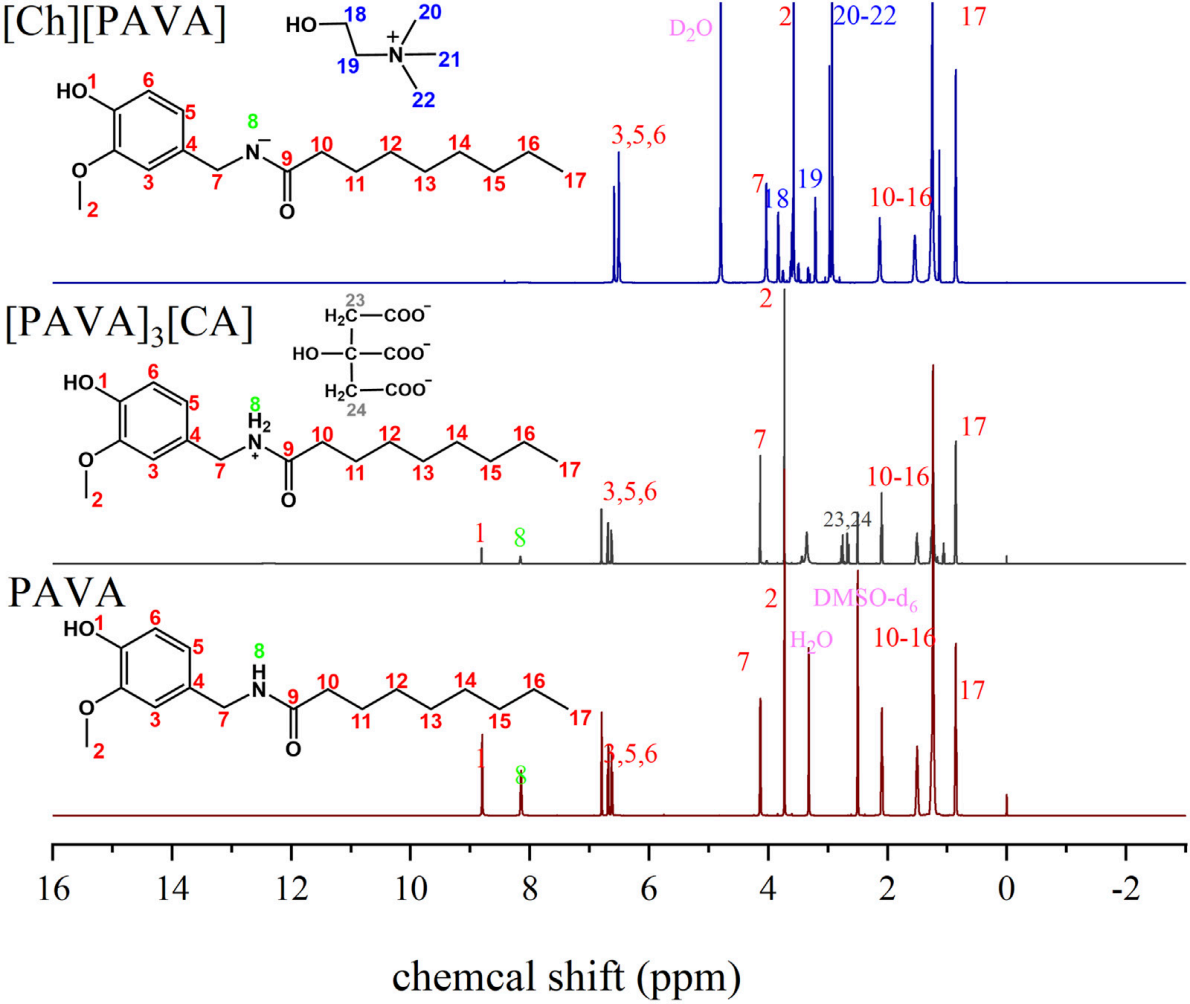
^1^H NMR of [Ch][PAVA], [PAVA]_3_ [CA] and PAVA.

Based on the comparison of the ^1^H NMR spectra of PAVA and [Ch][PAVA] (Figure 4), it can be clearly seen that the chemical shifts of 8.80 and 8.15, which respectively represent the phenolic hydroxyl (–OH) and amide (–NH–) groups in PAVA, were absent in the NMR spectrum of [Ch][PAVA]. This may be due to the reaction of–OH and–NH–or the activation of H on the functional groups, leading to disappearance from the spectrum. Additionally, we observed a certain bias in the shifts of other functional groups on PAVA, which may be attributed to the effects caused by cation and anion formation. In the NMR spectrum of [PAVA]_3_ [CA], in addition to the disappearance of the shifts of–OH, –NH–, and the carboxyl group (–COOH), there were less changes in the chemical shift of the other functional groups than the [Ch][PAVA]. A possible explanation is the presence of weaker interactions within [PAVA]_3_ [CA] than the [Ch][PAVA], which exerted smaller effects on the functional groups.

For [Ch][PAVA], 1H-NMR peaks 19 and 2 respectively represent N-CH_2_ and ArCH_3_ in [Ch]^+^ and [PAVA]^‒^ and the area proportion of them is 2:3, which show that the proportion of cation to anion is 1:1. For [PAVA]_3_ [CA], 1H-NMR peaks 2 and 24 respectively represent Ar-OCH_3_ and C-CH_2_ in [PAVA]^+^ and [CA]^3‒^ and the area proportion of them is 3:0.67, which show that the proportion of cation to anion is 3:1.

#### 3.1.3 Elemental analysis of the prepared compounds

The elemental analysis data of [Ch][PAVA] and [PAVA]_3_ [CA], including N, C, H, and O, is shown in Table 1. It can be seen that the measured data of element content for each ionic liquid is basically consistent with the theoretical values. Therefore, elemental analysis characterization can further demonstrate the proportions of cation:anion for prepared [Ch][PAVA] and [PAVA]_3_ [CA].

**TABLE 1:** Elemental analysis data of [Ch][PAVA] and [PAVA]_3_ [CA].

No.	Ionic liquids		N	C	H	O
1	[Ch][PAVA]	*w*_Measured_/%	6.47	64.56	10.07	18.68
		*w*_Calculated_/%	7.07	66.67	10.10	16.16
2	[PAVA]_3_ [CA]	*w*_Measured_/%	3.83	62.90	8.39	23.11
		*w*_Calculated_/%	3.92	63.87	8.31	23.90

#### 3.1.4 TGA and DSC characterization of the prepared compounds

**FIGURE 5:**
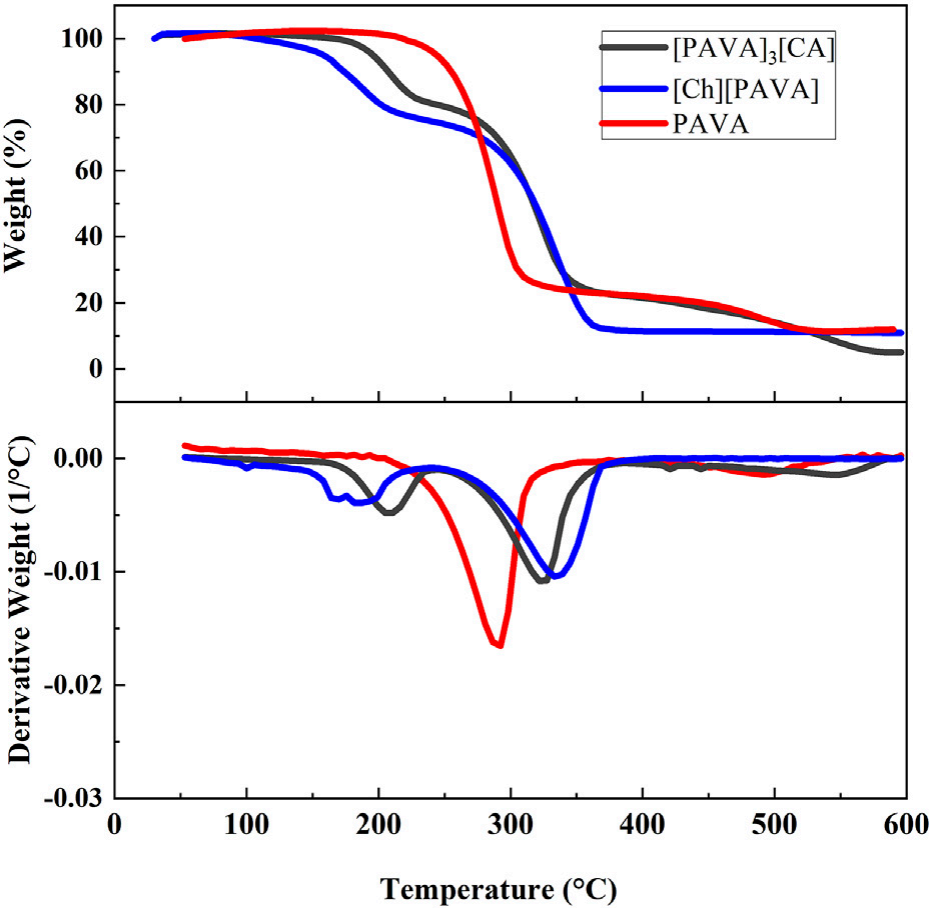
TGA graphs of PAVA, [Ch][PAVA], and [PAVA]_3_ [CA].

The TGA graphs indicate that [Ch][PAVA] and [PAVA]_3_ [CA] had relatively higher thermal stability, exhibiting decomposition temperatures (*T*_d_) of 181.98°C and 206.05°C, respectively (Figure 5). Compared with pure PAVA, the weights of [Ch][PAVA] and [PAVA]_3_ [CA] slightly decreased when the temperature increased from 100°C to 150°C because of water evaporation. This is mainly explained by the fact that salt–based ILs readily absorb moisture from the air. Below 600°C, both [Ch][PAVA] and [PAVA]_3_ [CA] exhibited two–step thermal decomposition within the temperature ranges of 150°C–250°C and 250°C–500°C. The first thermal decomposition step may be attributed to the generation of PAVA from the decomposition of the PAVA–based IL. This led to mass reductions of 26% and 20%, respectively, which were comparable to the mass fractions of choline and CA in the ILs. The second thermal decomposition step involved the decomposition of PAVA. Changes in the decomposition temperatures of PAVA revealed that PAVA decomposition temperatures after IL formation were 326.01°C and 336.94°C, respectively, which were both higher than that of single–compound PAVA (290.68°C). Therefore, it can be inferred that ILs of PAVA will exhibit better anti–thermal decomposition performance than single–compound PAVA during heat dissipation processes such as explosions.

### 3.2 Hydrophilicity of ILs

**FIGURE 6:**
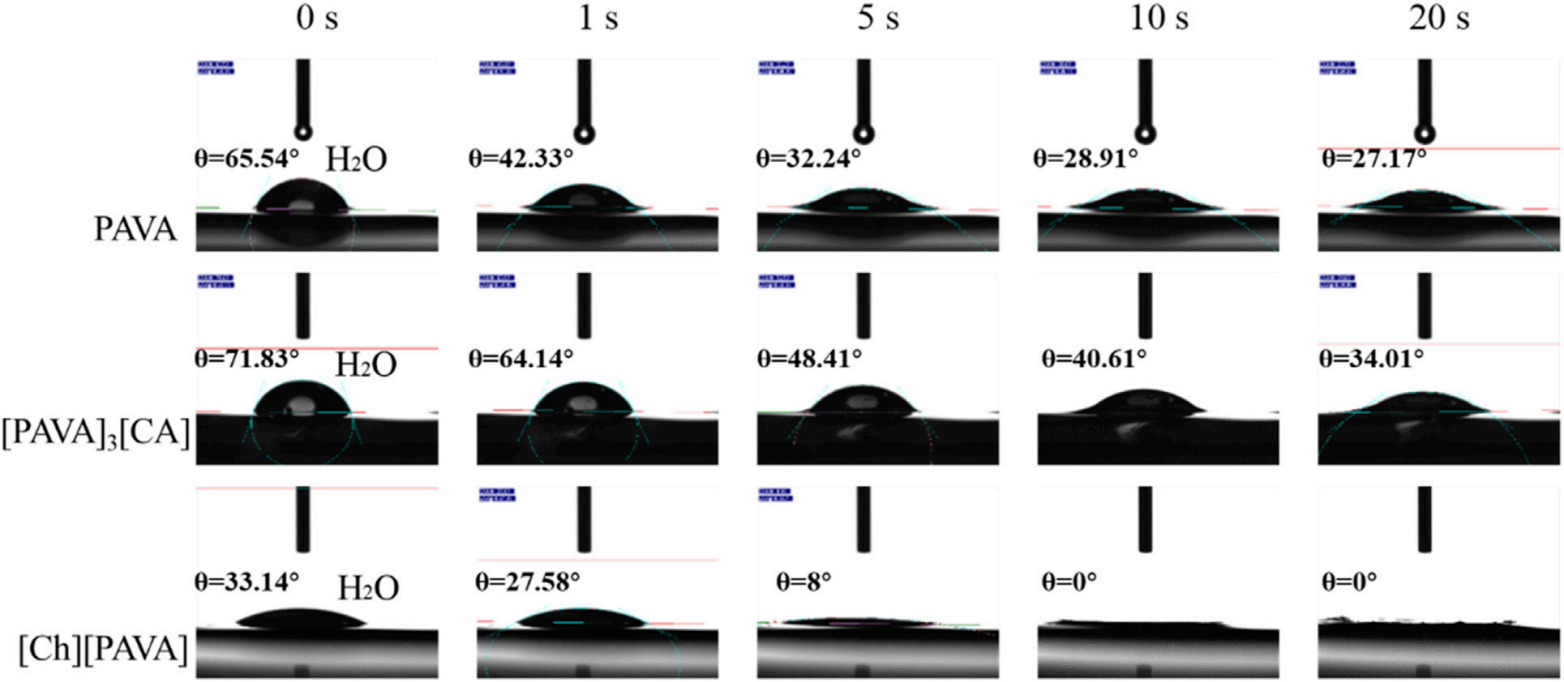
Digital image showing contact angles of water droplets on PAVA, [PAVA]_3_ [CA], and [Ch][PAVA].

**FIGURE 7:**
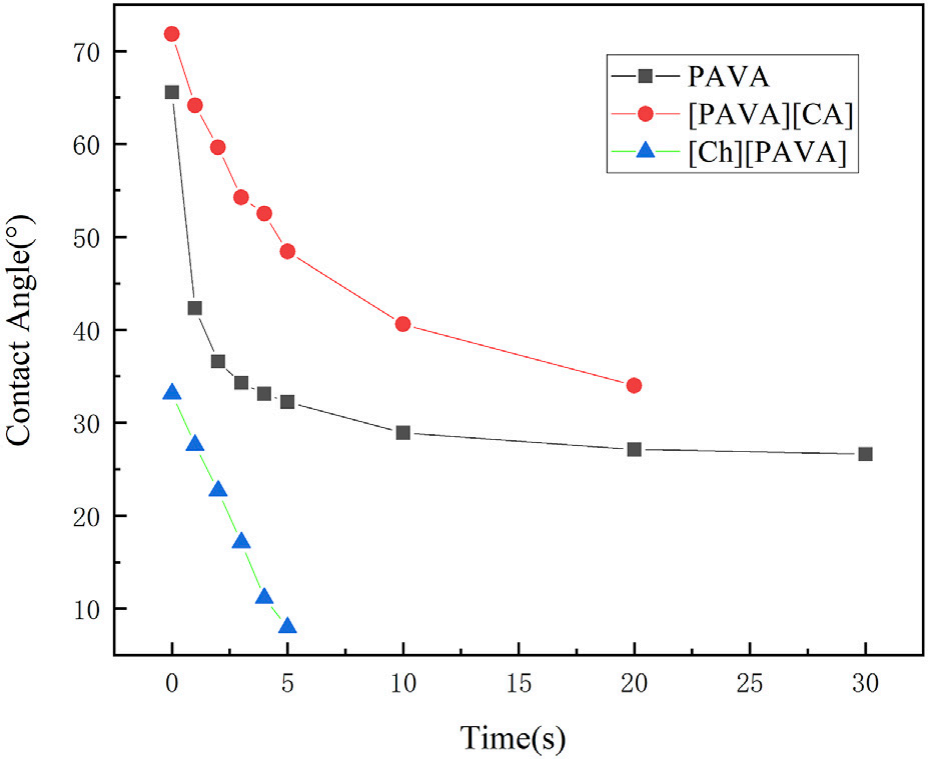
Changes over time in contact angles of water with PAVA, [PAVA]_3_ [CA], and [Ch][PAVA].

Contact angles were measured using water as the test liquid to determine the degree of hydrophilicity of the compounds. As shown in Figure 6, a large contact angle of approximately 65.5° was reached at 0 s for pure PAVA, which was mainly due to its strong hydrophobicity. PAVA has a relatively high molecular weight and cohesive energy and possesses a structure containing both hydrophobic octyl side chains and phenolic and amide groups that provide multiple sites for oxygen bond reactions. The unique backbone and functional groups of PAVA, combined with the complex inter–and intra–molecular interactions along with the spatial arrangement and configuration that they induce, contribute to the poor solubility of PAVA, with extremely low solubilities in water and organic solvents containing nonpolar or weakly polar simple molecules. However, the introduction of the strongly hydrophilic choline structure to form the [Ch][PAVA] IL resulted in a significantly smaller contact angle under identical conditions. The water contact angle of [Ch][PAVA] was approximately 33.1° at 0 s and reached 8° at 5 s, which was markedly lower than those of [PAVA]_3_ [CA] (approximately 71.8° at 0 s) and PAVA (approximately 65.5° at 0 s). Further analysis revealed that the water contact angle of [Ch][PAVA] decreased linearly in a time–dependent manner, with the water droplet completely penetrating the sample within 10 s (Figures 6, 7). These results demonstrate the high affinity of [Ch][PAVA] for water.

[Ch][PAVA] contains a quaternary ammonium cation structure, which has certain ionization and strong polarity. It can interact with polar solvents such as water, enhance intermolecular electrostatic forces, and form stronger hydrogen bonds. Therefore, [Ch][PAVA] containing a quaternary ammonium cation structure has significantly higher solubility in polar solvent water than [PAVA]. The water solubility of [PAVA]_3_ [CA] is worse than that of PAVA which maybe due to the hydrophilic groups of [PAVA]_3_ [CA] less than PAVA and CA and the hydrophilic groups of PAVA and CA formed hydrogen bonds or other interactions with each other. And this is maybe why CA used as a material for hydrophobic modification.

$$\log P=\frac{-\left(E\text{octanol}-E\text{water}\right)}{2.303RT}$$

The calculation results (Table 2) indicate that preparation of ILs using PAVA increased the water solubility of PAVA to a certain extent. It can be deduced that the distribution of PAVA in the aqueous phase was enhanced through electrostatic and hydrogen bond interactions after [Ch][PAVA] formation, which contributed to the increased hydrophilicity. The structure of [Ch][PAVA] shows the formation of cation–anion pairs between PAVA and quaternary ammonium salts, which enabled the continuous existence of [PAVA]^‒^. However, the solubility of [PAVA]_3_ [CA] is worse than PAVA, maybe resulting in a weakly hydrophilic and space effect of CA.

**TABLE 2:** Calculated lipid–water partition coefficients of PAVA, [Ch][PAVA].

No.	Compound	*E*_octanol_	*E*_water_	log*P*
1	PAVA	−944.997	−944.991	2.6550
2	[Ch][PAVA]	−1,273.45	−1,273.44	2.2263

**FIGURE 8:**
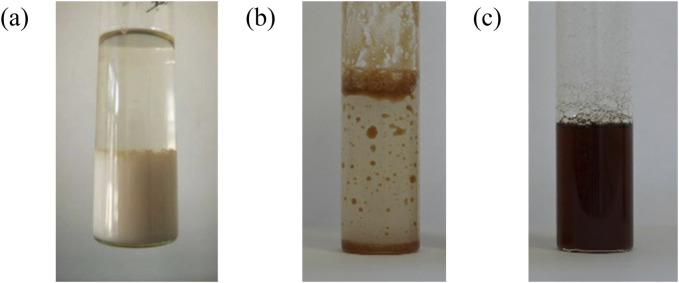
Dissolution of **(A)** PAVA, **(B)** [PAVA]_3_ [CA] and **(C)** [Ch][PAVA] (all with 10wt% PAVA).

Figure 8 shows that pure PAVA powder and [PAVA]_3_ [CA] were insoluble in water and formed significant sedimentation and stratification. However, [Ch][PAVA] was uniformly dispersed in water (Figure 8C) without separation, forming a transparent, stable solution. This result is highly consistent with the results of the contact angle experiment and that may be attributed to the strong hydrophilicity of [Ch]^+^.

**FIGURE 9:**
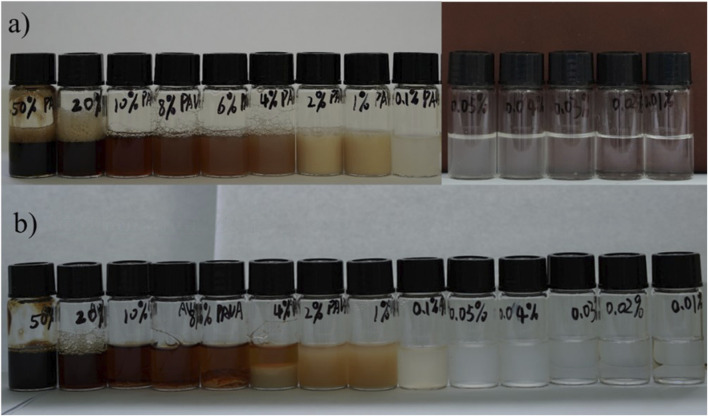
Dissolution of of [Ch] [PAVA] with different PAVA concentrations. **(A)** Newly prepared solution. **(B)** Solution after 72 h.

We also explored the solubility of [Ch][PAVA] with PAVA concentrations decreasing from 50wt% to 0.01wt%, and the results are shown in Figure 9. It can be seen that the 0.02wt% aqueous solution of PAVA remains clear and transparent, indicating that [Ch][PAVA] is much more soluble than PAVA powder in water. At the same time, the dissolution of [Ch][PAVA] in water showed good solubility at both low and high concentrations. This interesting phenomenon may be the result of competition interaction between [PAVA]^‒^ and water and it can be explained set on excess enthalpy. The reported experimental data state that the thermodynamic quantities of mixing could provide valuable insight into molecular interactions and microscopic structures in such mixtures (Ficke et al., 2010; Podgoršek et al., 2016; Ma et al., 2018). And some (ionic liquids + water) mixtures show an S-shaped curve dependence of Δ_mix_ *H* with composition with positive enthalpies of mixing for compositions poor in ionic liquids. When the concentration of [Ch][PAVA] is high, the interaction between [Ch]^+^ and [PAVA]^−^ is strong and cannot be completely destroyed, the H_2_O molecules mix well with IL with strong interactions (van der Waals, H-bonds, electrostatic, etc.), and H_2_O is embedded inside and at the periphery of the IL without any H_2_O clusters formed and it likes water soluble in [Ch][PAVA]. With a further addition of H_2_O, the IL clusters start to dissociate into individual ions or ion-pairs and the dissociated ions are gradually hydrated. When the concentration of [Ch][PAVA] is extremely low, the IL structures are completely dissociated as ions. Depending on the size of the IL-ions, some of them are fully hydrated, while some are partially hydrated and [Ch]^+^ are surrounded by water molecules, greatly weakening the interaction of [Ch]^+^ and [PAVA]^‒^, which resulting in poor solubility of ionic liquids in the system.

### 3.3 Irritation effects

**FIGURE 10:**
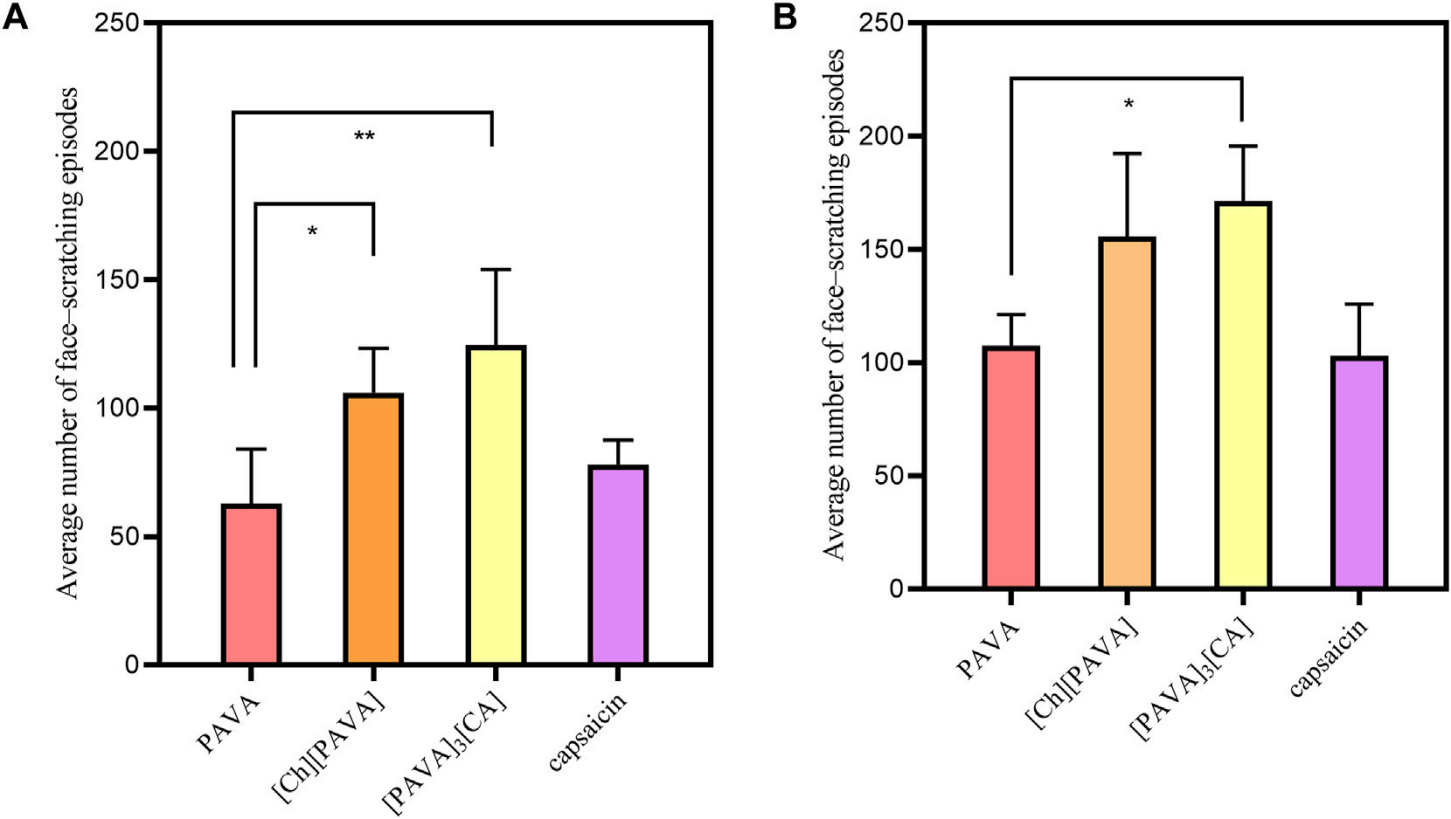
Results of animal irritation experiment within 1 min **(A)** and 5 min s **(B)**.

As shown in Figure 10, [PAVA], [Ch][PAVA], [PAVA]_3_ [CA], and capsaicin all have strong irritant responses in the animals, which included violent face–scratching, squinting, sneezing, and tachypnea. The experiment observed two time periods of 1 min and 5 min, and the number of face scratching episodes observed in the animals were statistically analyzed. Within 1 min, with PAVA sample as the control group, there was a significant difference between the [Ch][PAVA] sample (P < 0.05) and the [PAVA]_3_ [CA] sample (P < 0.05), while there was no significant difference in the capsaicin sample (P > 0.05). Within 5 min, there was a significant difference between the [PAVA]_3_ [CA] sample (P< 0.05), while there was no significant difference between the [Ch][PAVA] sample (P > 0.05) and the capsaicin sample (P > 0.05). Compared with the PAVA and capsaicin samples at identical concentrations, the [Ch][PAVA] and [PAVA]_3_ [CA] samples exhibited irritant effects that were comparable or even stronger.

## 4 Conclusion

In this study, we proposed a method for the introduction of readily water–soluble ions into PAVA to form ILs. Two different types of ions, namely [Ch]^+^ and [CA]^3‒^, were introduced to compare the effects of different ions on PAVA properties. The [Ch][PAVA] IL exhibited the best dissolution performance among the various test compounds. Our results indicated that the introduction of highly water–soluble choline cations into the PAVA IL improved the hydrophilicity of PAVA. Upon the formation of an IL, the distribution of PAVA in the aqueous phase could be improved through the presence of electrostatic and hydrogen bond interactions, thereby enhancing its hydrophilicity. When diluted solutions of the prepared ILs were used in an animal irritation experiment, the synthesized ILs exhibited irritant effects comparable to those of PAVA and natural capsaicin when using identical concentrations. Therefore, the PAVA–based ILs synthesized in this study may potentially serve as a promising novel riot control agent.

## Data Availability

The original contributions presented in the study are included in the article, further inquiries can be directed to the corresponding author.
